# In-and-Out of Tobacco Farming: Shifting Behavior of Tobacco Farmers in Indonesia

**DOI:** 10.3390/ijerph17249416

**Published:** 2020-12-15

**Authors:** Gumilang Aryo Sahadewo, Jeffrey Drope, Qing Li, Firman Witoelar, Raphael Lencucha

**Affiliations:** 1 Faculty of Economics and Business, Universitas Gadjah Mada, Sleman 55281, Indonesia; gasahadewo@ugm.ac.id; 2 Health Policy and Administration, School of Public Health, University of Illinois at Chicago (UIC), Chicago, IL 60608, USA; 3 Economic & Health Policy Research, Data Science, American Cancer Society, Atlanta, GA 30303, USA; qing.li@cancer.org; 4 Crawford School of Public Policy, Australian National University, Canberra 2600, ACT, Australia; firmanwitoelar.Kartaadipoetra@anu.edu.au; 5 Faculty of Medicine, School of Physical and Occupational Therapy, McGill University, Montreal, QC 3605, Canada; raphael.lencucha@mcgill.ca

**Keywords:** tobacco farming, farming decisions, alternative livelihoods, Framework Convention on Tobacco Control, longitudinal survey, focus group discussion, multinomial logit regression

## Abstract

Understanding the variables that affect farmers’ decisions as to whether to grow tobacco and/or other crops provides important insights into their economic lives and can help to inform the development and implementation of policies that shape both tobacco production and tobacco control, such as increasing tobacco excise taxes. This study employs complementary quantitative and qualitative methodologies to identify variables that affect tobacco farmers’ economic decision making in Indonesia, a major tobacco producer. The research focuses on the variables that affect tobacco farmers’ decisions to continue tobacco farming or shift to non-tobacco farming. It finds that tobacco farmers’ decision making is complex but also predictable. The results of the quantitative analysis suggest that farming profits and positive rainfall shocks are two of the key variables that affect the decision to cultivate tobacco. The qualitative results confirm these findings and further illuminate that access to credit, education (agricultural and otherwise) and information play substantial roles in farmers’ economic decision making. Most of these variables are affected by the unequal relationship between the tobacco firms that buy tobacco and the farmers, wherein the farmers are consistently at a disadvantage in terms of negotiating key parameters such as prices and evaluation of leaf quality.

## 1. Introduction

In the last five years, research has documented empirically that in many low- and middle-income countries (LMICs), tobacco farming is rarely an economically lucrative endeavor for smallholder farmers [1,2,3,4]. Yet farmers across LMICs continue to grow tobacco and in some countries, tobacco production is increasing. Often tobacco companies incentivize production by providing credit, inputs, and other resources that farmers would not otherwise be able to access [5,6]. The nature of tobacco production is also changing with the increasing use of often precarious contractual relationships between farmers and tobacco companies [7,8]. Tobacco farming also remains one of the most labor-intensive crops, often driving cash-poor farmers to employ their own and other children [9], and causes green tobacco sickness, a form of acute nicotine poisoning, among many farmers [10]. Given the challenges of tobacco growing, policy makers, advocates, and farmers have sought alternatives to tobacco production. As part of this scenario, evidence from Indonesia suggests a complex scenario wherein many farmers shift in and out of tobacco production, but very little is understood about the variables that shape these decisions.

Though typically among the top five tobacco leaf producers globally, tobacco is a proportionally small economic sector in Indonesia, generally comprising less than 0.3% of the total value of agricultural production and about 0.03% of gross domestic product [11]. Most tobacco leaf is used by the domestic industry for manufacturing kreteks, the popular Indonesian cigarette that combines tobacco and clove leaves, and though it typically exports small amounts each year, Indonesia is a net importer of tobacco leaf. The cigarette manufacturing sector is dominated by a small number of large firms that wield enormous amounts of well-documented political power [12]. The related tobacco farming sector derives its political power not only indirectly from the manufacturers, but also because the approximately 500,000 farming households are concentrated mostly in a small number of districts in Central and East Java.

The Framework Convention on Tobacco Control (FCTC) obligates Parties to help those working in the tobacco industry to transition to other viable alternative economic livelihoods, so an understanding of the variables that shape farmers’ major economic decisions, including shifting away from but also back to tobacco, will be instrumental in designing programs to help these farmers. This latter consideration is particularly important for countries that are presently heavily invested in supporting tobacco production, one of the reasons Indonesia has not yet signed the FCTC [13]. To date, the broader political economy of tobacco policy and market structure in countries have been the emphases of tobacco farming scholarship. Only recently has a robust body of literature emerged from a tobacco control perspective analyzing the factors that shape farmers’ decisions at the household level. Understanding the variables involved in household-level decision-making and in the perspectives of tobacco farmers provides a unique vantage point from which to understand the opportunities and challenges that they face, both with tobacco growing and the pursuit of alternatives.

This research employs complementary quantitative and qualitative methodologies to illuminate the main variables shaping farmers’ economic decision-making. The quantitative analysis is based on a longitudinal survey of current and former tobacco-farming households in Indonesia, whereas the qualitative component draws from focus group discussions (FGDs) in several major tobacco-growing areas in Indonesia. The purpose of this analysis is to examine the factors that influence the farmers’ major economic decisions, particularly which crops to grow but also whether to pursue other non-agricultural economic opportunities.

Theoretically, this research follows three complementary approaches. First, the extant literature cited above consistently demonstrates that micro-economic conditions—particularly the imperative to provide economically for the household—greatly affect the overall logic of farmers’ decisions to grow specific crops and/or engage in other economic activities, even if their perceptions and the revealed reality do not necessarily match. Accordingly, we examine the effects of these variables and, by employing complementary quantitative and qualitative methodological approaches, we compare the revealed (through the survey) and the perceived (through the FGDs). Second, and closely related to the first, farmers’ economic decisions are shaped by their institutional contexts, including such features as the availability of credit, information and education. We therefore examine these structural constraints and opportunities. Finally, because there is a decades-long history of tobacco growing in Indonesia, we also examine the effects of non-economic factors such as the tradition in many families of growing tobacco.

## 2. Materials and Methods

### 2.1. Tobacco Farmer Survey: Sample

Our sample consisted of farmers with experience of growing tobacco either currently or in the past. There were two waves of data collection. The first wave of the survey was fielded at the end of 2016 in seven top tobacco-producing districts. These districts include Magelang, Temanggung (Central Java province), Lumajang, Jember, Bojonegoro (East Java province), Lombok Tengah, and Lombok Timur (West Nusa Tenggara province). We obtained a total sample of 1350 households for the wave 1 survey. Drope et al. [14] provide a detailed discussion on sample size determination and the survey instrument.

The second-wave survey was fielded at the end of 2017 using the same survey instrument used in the first wave survey. Only a subset of the baseline sample was followed up for Wave 2 owing to cost considerations of survey implementation. Wave 2 was implemented in the Javanese districts which represent about 90% of total tobacco farming households in Indonesia. The households in West Nusa Tenggara (approx. 10% of tobacco farming households) were not revisited due to the same cost constraints. A total of 660 farming households were re-interviewed in the second-wave survey. Sahadewo et al. [15] provide a detailed discussion on the re-sampling and re-interview protocol for the Wave 2 tobacco farmers survey.

We keep households who are observed in both survey waves for the quantitative analysis. We summarize the composition of the sample in Table 1.

### 2.2. Comparing Groups of Farmers

We test differences in distributions of selected variables between farmer groups. Specifically, we compare either between tobacco farmers who stayed farming tobacco and tobacco farmers who shifted out of tobacco farming, or between non-tobacco farmers who stayed farming non-tobacco crops and non-tobacco farmers who shifted into tobacco farming. Selected variables that we investigate include household characteristics and farming outcomes. We test whether distributions of observations obtained between the two different groups on the selected variable are systematically different using the Wilcoxon rank-sum test.

We also conduct two-way tabulations between farmer groups and categorical variables such as whether farmers considered switching or reasons why farmers opted out of tobacco farming. We test whether there is an association between the two variables using the Pearson’s Chi-squared test.

### 2.3. Regression Analysis on Determinants of Farming Decisions

We conduct a regression analysis to estimate determinants of farming decisions among tobacco and non-tobacco farmers. Specifically, we analyze whether households’ characteristics and farming outcomes in the first wave can predict farming decisions in the second wave of the survey. Non-tobacco farmers may choose to stay farming non-tobacco crops or shift into tobacco farming. On the other hand, tobacco farmers may choose to stay farming tobacco or shift out of tobacco farming. Thus, there are four farming outcomes that we observe in the data: stay farming non-tobacco crops (*stay former*), stay farming tobacco crops (*stay current*), shift into tobacco farming (*into tobacco*), and shift out of tobacco farming (*out of tobacco*).

Given the categorical nature of the outcome variable, we utilize a multinomial logistic model to estimate the relationship between determinants and the farming decisions. Let Pr(*Y_i_*,_*t*_ = *m*) be the probability that we observe farming outcome *Y* for household *i* at period *t* equal to a specific outcome *m*. Let G be the logistic function. The multinomial logistic model is:

Pr(*Y_i,t_* = *m*) = G(***X_i_***,_***t***−1_) (1)

where *m* = {*stay former*, *stay current*, *into tobacco*, and *out of tobacco*} and ***X_i_***,_***t***−1_ is a vector of variables which include households’ characteristics and farming outcomes in the first wave of the survey. These characteristics include head of household’s age, years of schooling, marital status, and household size. We also include the log of household assets, defined as the estimated current values of appliances, equipment, livestock, agriculture, and farming goods. Farming outcome variables include profit per hectare, the log of agricultural wage income, the log of non-agricultural wage income, the log of cultivated area, and share of cultivated land for tobacco farming. We observe that a significant share of tobacco farmers has tobacco farming contracts. Therefore, we include an indicator of whether a tobacco farmer has a tobacco farming contract.

We also include rainfall deviation from the long-run average during the previous tobacco farming season as one of the explanatory variables. The inclusion is important because relatively dry conditions in the pre-harvest period led to better tobacco farming outcomes [12,16]. A recent study also shows that rainfall deviation from the long-run average during the previous tobacco farming is significantly correlated with share of land allocated to tobacco farming [3]. Lastly, we include region dummies to control for region-specific unobserved factors that affect farming decisions. We investigate the variance inflation factors of the explanatory variables, and all of the variance inflation factors are below 4. This suggests that there is no multi-collinearity issue among the explanatory variables.

We report average marginal effects which indicate the change in the likelihood of a particular outcome as a response to a unit change in the explanatory variable holding all else constant. The estimated coefficients from the multinomial logistic regression do not reflect the marginal effect of the change in corresponding regressors as in an ordinary least squares regression. The marginal effect of the change in a particular regressor exhibits a scaling factor, which is a function of the explanatory variables ***X_i_***_,***t***−1._ and is unique for each unit of observation. Given the estimated coefficients, we calculate marginal effects of each explanatory variable for all observations. We then take the average to obtain average marginal effects of each explanatory variable for each outcome *m*. The general interpretation of an average marginal effect of variable *k* is the effect of a change in *k* on the probability of an outcome *m.*

We use Stata version 15.1 (StataCorp LLC, College Station, TX, USA) to obtain the descriptive statistics and to conduct the regression analysis. We use the function *mlogit* to obtain coefficient estimates using the multinomial logistic regression. We then use the function *margins*, dydx(*) to obtain average marginal effects of each variable. We use heteroscedasticity robust standard errors for estimation of the standard errors to accommodate the general form of heteroscedasticity [17].

### 2.4. Focus Group Discussions (FGDs)

To complement the survey data, we implemented a series of FGDs shortly after the Wave 2 survey to capture contextual information beyond the survey and to help clarify issues that came to light from an initial analysis of the survey results. These FGDs were particularly focused on farmers’ economic decision making.

We implemented a set of five focus group discussions (FGDs) with both current and former tobacco farmers (*n* = 34). We selected the villages using purposive sampling, choosing communities with significant tobacco growing (i.e., a significant percentage of arable land allocated to tobacco growing) in the same sub-districts where we implemented the survey. Between February and March 2019, we conducted the FGDs in three of these same districts. In the village of Wonoroto (Windusari sub-district of Temanggung district), we conducted an FGD with five former tobacco farmers. Separately, we held an in-depth interview with the leader of the local farmers’ group. In Lumajang district, we conducted two FGDs: a group of eight current tobacco farmers (village of Bades, Pasirian sub-district), and a group of five former tobacco farmers (village of Pandanwangi, Tempeh sub-district). In Bojonegoro, we conducted two FGDs: eight current tobacco farmers (Woro village, Kepohbaru sub-district), and eight former farmers (Setren village, Ngasem subdistrict).

The survey team supervisor responsible for the study area selected the participants from a list provided by village leaders and farmers. The supervisor evaluated the eligibility of the participants: current or former tobacco farmer (and not a village official). Experienced facilitators with backgrounds in qualitative research led the FGDs in the Indonesian language and local dialect (Javanese/East Javanese), actively encouraging different perspectives and avoiding dominance of any specific individuals.

The participants were guided to reflect upon and actively discuss the following: overall experience with tobacco farming, how tobacco leaf is graded and priced, input costs, the labor demands of different types of farming, the profitability of tobacco farming, and other important issues identified by the farmers. Those farmers currently growing tobacco were asked to discuss the importance of tobacco farming relative to their other economic activities, and any consideration given to shifting to other crops. Former tobacco farmers were asked to reflect on their previous tobacco farming experiences, the reasons they stopped cultivating tobacco, and any consideration given to shifting back to tobacco. The FGDs were by design open and free-flowing and, though the facilitator broadly introduced the above themes (see Appendix A for the list of overarching questions), the discussions were effectively led by the participants.

The FGDs were recorded, transcribed verbatim, and translated into English. Analysis of the FGDs utilized the transcripts and other notes from the FGDs, including participation level of each participant, seating location of the participants, and farmers’ engagement in the exercise. Two of the investigators (J.D. and Q.L.) independently identified key topics and sub-topics related to shifting behaviors from the original guiding questions and, reading through the transcripts, identified new significant relevant themes that emerged in the FGDs. Using these themes, an experienced research assistant, with regular quality assurance from the two investigators (JD and QL), grouped these “chunks” of data from all of the transcripts using MAXQDA 2020 qualitative software (VERBI GmbH, Berlin, Germany). The investigator team discussed the findings to reach mutual understanding of the data. The study protocol received ethics approval from the Institutional Review Boards of SurveyMeter in Indonesia and the Morehouse School of Medicine (the IRB of record for one of the research’s co-sponsors, the American Cancer Society).

## 3. Results

### 3.1. Shifting Status

We summarize the distribution of farmers by cross-wave farming decisions in Table 2. In particular, there are four types of farming households: farmers who did not farm tobacco in both waves (14.09%), farmers who farmed tobacco in both waves (66.21%), farmers who shifted into tobacco farming (11.36%), and farmers who shifted out of tobacco farming (8.33%). We note that there was a non-negligible share of farmers who switched into and out of tobacco farming. The proportion of 2016 tobacco farmers who stopped (11%) was smaller than the proportion of 2016 non-tobacco farmers who shifted into tobacco farming (45%).

We note that most farmers who shifted out of tobacco farming are still farming (85.45%). In addition, farmers who shifted out of tobacco farming also obtain livelihood from other sources such as business enterprises and wage employment.

In Table 3, we present results from an analysis of whether current tobacco farmers considered switching in 2016 and their actual farming choice in 2017. From elsewhere in the survey, about 7% of current tobacco farmers in 2016 considered shifting out of tobacco farming. Among current tobacco farmers who considered shifting out of tobacco farming in 2016, about 24% eventually shifted out of tobacco farming. On the other hand, only about 10% of current tobacco farmers who did not consider shifting out of tobacco farming in 2016 eventually shifted out of tobacco farming in 2017. The Pearson Chi-squared test shows that we reject the null hypothesis of independence. This result is evidence that considering shifting out of tobacco farming is an important explanation of a shifting out decision.

In Table 4, we tabulate reasons why former tobacco farmers opted out of tobacco farming in 2016 and farming choice in 2017. The low selling price of tobacco leaf and weather are two of the popular reasons why farmers opted out of tobacco farming in 2016. Among those who stayed farming non-tobacco crops in 2017, about 35% argued low price was the reason. The share of former tobacco farmers who indicated lower price as a factor associated with shifting is higher than the share of former tobacco farmers who shifted back to tobacco farming in the second wave (20%). This suggests a complex dynamic wherein there is selection on the decision to farm tobacco—i.e., it is partly endogenous (correlated with other variables). Specifically, former tobacco farmers who switched back to tobacco farming were less likely to be affected by the low selling price of tobacco leaf.

Weather is associated with former tobacco farmers’ decision to switch out of tobacco farming in 2016. The 2016 weather affected more former tobacco farmers who shifted back to tobacco farming in 2017 than those who stayed farming non-tobacco. We found from our focus group discussions that weather is one of the contributing factors of tobacco farming. Sahadewo et al. [15] also show that weather was one of the top three reasons for growing tobacco in 2017. Another explanation is that they may have had an expectation of weather well suited for tobacco growing in 2017 after the relatively bad (very wet) weather in 2016. A study shows that unfavorable weather in 2016 is associated with a larger share of land for tobacco farming, which is an indication of such expectation [3].

### 3.2. Farmers’ Characteristics Based on Shifting Status

We summarize households’ characteristics and farming outcomes in 2016 by farming choice in 2017 in Table 5. The following analysis aimed to identify whether there are differences in socio-economic characteristics and farming outcomes between former and current farmers, and between those who shifted in and out of tobacco farming.

In general, tobacco farmers who continued to farm tobacco are more experienced (Wilcoxon rank-sum, *p*-value < 0.001) and have more assets than those who shifted out of tobacco farming (Wilcoxon rank-sum, *p*-value < 0.10). Tobacco farmers who continued to farm tobacco also dedicated a higher share of their land for tobacco farming (Wilcoxon rank-sum, *p*-value < 0.05). On the other hand, tobacco farmers who shifted out of tobacco farming dedicated a higher share of their land for non-tobacco farming (Wilcoxon rank-sum, *p*-value < 0.10).

These differences are also reflected in farming outcomes in the first wave. The average land cultivated by current tobacco farmers (12.23 hectares) is three times the average land cultivated by farmers who shifted out of tobacco (4.24 hectares), and the difference is statistically significant (Wilcoxon rank-sum, *p*-value < 0.01). Consequently, tobacco yield and tobacco sales between these two groups were quite different. In particular, the average tobacco yield by current tobacco farmers (539.3 kg) was far larger than the average tobacco yield by farmers who switched out of tobacco farming (165.7 kg). The difference is statistically significant (Wilcoxon rank-sum, *p*-value < 0.001). Consequently, the average tobacco sales among current tobacco farmers is three times that of farmers who switched out of tobacco farming in 2016.

We also observe that farmers who switched out of tobacco generated lower tobacco sales (Wilcoxon rank-sum, *p*-value < 0.001) and non-tobacco crop sales, particularly in the non-tobacco season (Wilcoxon rank-sum, *p*-value < 0.01).

We find that former tobacco farmers who continued to farm non-tobacco crops and those who shifted into tobacco farming had similar socio-economic characteristics and farming outcomes in 2016. There were no significant differences between these two groups of farmers in terms of assets, land cultivated, crop income in the wet and dry season, or agricultural and non-agricultural wage incomes (Wilcoxon rank-sum, *p*-value > 0.10 for all tests).

Interestingly, we find that in 2016 former tobacco farmers who shifted into tobacco farmers had higher income per hectare than those who stayed farming non-tobacco crops. The difference in income per hectare is statistically significant (Wilcoxon rank-sum, *p*-value < 0.05).

These results so far suggest that there is a selection among those who stayed as tobacco farmers and those who switched out of tobacco farming. Those who stayed as tobacco farmers relied more on their tobacco crop and have been farming tobacco for a long time. On the other hand, farmers who switched out of tobacco farming were more reliant on non-tobacco and non-farming income.

There were not many differences between former tobacco farmers who stayed farming non-tobacco crops and those who shifted into tobacco farming. However, farmers who went into tobacco farming generated slightly larger income per hectare, driven by larger crop income in the wet (non-tobacco) season. Farmers who switched back to tobacco farming may have used some of the profits as capital for tobacco farming, as capital was cited as one of the reasons farmers opted out of tobacco farming.

### 3.3. Determinants of Shifting Behavior

The result of the regression analysis presented in Table 6 largely confirms findings from the descriptive analysis. Former farmers who generated positive profits in the first wave of the survey were associated with a significantly higher likelihood of shifting into tobacco farming. We also find that weather shocks in the previous year play a significant part in determining the choice of tobacco farming in the current year. In particular, current tobacco farmers who experienced higher-than-average rainfalls by one standard deviation in the previous period had a higher probability of staying as tobacco farmers and had a lower probability of shifting out of tobacco farming.

Higher-than-average rainfalls during tobacco farming season are not preferable, because tobacco farming requires relatively dry conditions, particularly close to the harvest period [12]. The main explanation of this finding is that farmers expect more suitable weather for tobacco farming after a particularly wet year. This finding is consistent with a previous finding that positive rainfall shock in the previous period was positively correlated with the share of land allocated to tobacco farming in the following year [3].

There is also a notable correlation between having a contract and farming decisions. In general, having a tobacco farming contract had a positive association with tobacco farming decisions among current tobacco farmers in wave 1. This result is sensible because none of the former tobacco farmers in wave 1 had a tobacco farming contract in wave 1. The relationship between having a tobacco farming contract and farming decisions was stronger among farmers who stayed as current tobacco farmers in both waves than among farmers who shifted out of tobacco in wave 2. This result is quite logical because stay-current farmers may be more reliant on their contractors for farming inputs and sale of tobacco leaf.

### 3.4. Results from Focus Group Discussion (FGDs)

We identified six clear themes from the analysis of FGDs. These themes shed light on shifting out of tobacco growing. These themes largely hew to the findings of the descriptive and regression results, suggesting that a complex but also identifiable set of narratives about shifting is emerging. In Table 7, we drew from the dozens of suitable direct quotes for these themes from the FGDs to provide samples of the nature of the farmers’ comments, paying attention to presenting multiple points of view where applicable.

By far the most commented-upon issue that farmers chose to discuss across districts was the influence and role of the tobacco industry. In particular, the farmers consistently noted the nature of the relationships that they had with tobacco companies and the overwhelming conclusion of most farmers was the consistent lack of fairness and a power dynamic that gave the tobacco companies significant advantages over the farmers. Several key sub-themes emerged here: contracts; grading; prices; and what was essentially the market structure in terms of how the farmers sold their tobacco leaf. Tobacco farmers consistently reported that the one thing they liked about the contracts with tobacco companies was that the companies provided inputs on loan, which did not require any initial capital to begin the growing season. They noted how vital this was to their household because access to credit for other agricultural (and non-agricultural) endeavors was weak (discussed below).

The farmers noted consistently the industry’s leaf purchasing practices. First, for non-contract farmers (who are obligated to sell their leaf to their contractor), there were many comments about how the companies manipulate the market by rotating which leaf buyer is buying in a given period of time. Very often there are only one or two buyers operating when the farmers attempt to sell their leaf, which means there is little or no competition and the farmers essentially must accept the price that they are offered (or not sell). Many noted that a few weeks later a particular buyer will stop buying and another will begin. There was a firm belief among the farmers that this depresses prices.

On a closely related note, many farmers commented that the grading process was rarely transparent. Many farmers had been cultivating leaf for many years, even decades, and maintained that they could identify the grade/quality of their leaf very readily. However, they complained that the grade assigned to their leaf by the buyers was very often well below the quality they knew their tobacco to be. Since there were no other buyers and they needed to pay back any loans and/or generate cash to pay household expenses, they were pressured into accepting these grade assignments, which had a negative effect on the prices they received.

Farmers focused a great deal on the economics of tobacco farming, which generally meant the level of profitability of growing the crop. Notably, there was very mixed feedback on this topic. Some farmers believed that tobacco cultivation is profitable, even one of the most profitable crops. Yet, many other farmers had the opposite view and complained that it was rarely profitable. Most farmers agreed that the variability of profit was wide, and weather was an important factor in determining profits (discussed below).

Almost all farmers agreed that access to credit or capital was an ongoing major concern and that this affected their decision to grow tobacco. Many farmers reported insufficient capital just before or at the beginning of the dry season to plant crops and when they looked for credit it was easy or at least easier to obtain credit to grow tobacco through contracts. Most farmers reported that credit for other crops or other off-farm economic activities was almost non-existent in their regions. In other words, the decision to grow tobacco was a direct result of both their lack of capital and the difficulty of obtaining credit for other economic activities. Notably, in a related dynamic, some farmers reported that when they had sufficient capital from the previous seasons (tobacco and/or non-tobacco), it motivated them to grow more tobacco.

Perhaps, as with most farmers throughout the world, and particularly where there is no irrigation and crops are principally rain-fed, the weather was a dominant discussion topic. The discussion was mainly about the cycles of weather and that a dry season, which is better for tobacco growing, typically followed a very wet one. The principal challenge that farmers identified was attempting to accurately guess when a season was going to be wet after a couple of drier years. These conjectures about weather fundamentally shaped the proportion of land that farmers allocated to growing tobacco and explains why there is very often a higher allocation to tobacco after a less profitable wet tobacco season.

The results of the FGDs reveal that tobacco has a deep multigenerational foothold among many communities. Some farmers referred to it as “part of the culture” broadly speaking or, more commonly perhaps, specific to their “family tradition.” Farmers’ parents and grandparents often also grew tobacco so it was almost a foregone conclusion that tobacco would be a significant part of their crop portfolio. Farmers consistently noted that they “knew how to farm tobacco” and had less experience growing other cash crops.

In a related finding from the FGDs and more relevant to their interaction—or a lack thereof—with government, farmers observed consistently in these discussions that there were essentially no agricultural extension services—community- or farm-level programs to help farmers increase yields and crop quality—for other crops from any level of government. They did, however, note that the tobacco companies had knowledgeable representatives in the field sharing information about tobacco growing techniques and related topics.

Finally, farmers placed a notable emphasis on information. Specifically, farmers observed consistently that it was difficult to obtain information about marketing their non-tobacco crops. They did not, for example, have ready access to market prices for other agricultural products. In contrast, the farmers reported that the tobacco companies made information readily available to farmers and that they found the relative certainty of tobacco growing to be reassuring even though they also knew that it was very unlikely to be lucrative in most years.

## 4. Conclusions

This research strongly reinforces that shifting behaviors of tobacco farmers are complex but also relatively predictable. There are multiple reasons that farmers choose to cultivate tobacco in a given year and multiple reasons that the same farmers might elect to grow other crops or engage in off-farm activities the next year. The broad theoretical framework proposed in the introduction posits that it is not just microeconomic conditions that shape farmers’ decisions to grow tobacco but their experiences and those of their families and communities historically. These might be described as tradition or culture, but they are clearly less obviously economic. The results also reveal that broader institutional structures shape farmers’ decisions because poor access to credit, education and/or information constrains their available options, real and perceived.

One explanation derived from the descriptive analysis is that those farmers who continue to grow tobacco have been farming tobacco for a longer period. The relative contentment with tobacco, stemming for many from the generational inheritance of tobacco growing, and a corresponding lack of knowledge of and experience with other crops, contributes to the perpetuation of tobacco growing. It may be financially difficult for these farmers to shift out of tobacco or these farmers may perceive that it is difficult to shift away.

Similarly, the findings, perhaps not surprisingly, indicate that tobacco is viewed as a lucrative crop and this reflects the findings that tobacco farmers sometimes earn more than non-tobacco farmers [3]. Additionally, tobacco farmers who fared poorly in the first wave of the study held onto the belief that staying with tobacco was the best economic decision. The decision-making often appears to be based on their aspiration that tobacco will be lucrative in the next season even in the face of a season of economic loss.

Education and information seem to shape farmers’ decisions and are features of the environment that have feasible potential for change. There appears to be a dearth of both for non-tobacco crops. Research on farmers in tobacco-growing regions of Kenya demonstrated that something as simple as reliable mobile phone service helps farmers to make better cropping decisions [18]. During economic restructuring in the 1980s and 1990s, many governments pared back agricultural extension services and this research suggests that a consequence of this decision is an agricultural workforce not well enough trained to diversify crop portfolios in order to minimize risk and maximize returns. Though farmers cannot obviously change the weather, having more information is likely to help them make better cropping decisions. On the supply side, the government could spend more resources providing this information to these communities. Government retrenchment in agriculture has allowed multinational companies and related firms to occupy the space, thereby encouraging crops that do not benefit the health or wealth of the local people.

Credit is a clear stumbling block to generating new economic activities for farmers. It is a lack of credit that appears to deeply condition a farmer’s decision to grow tobacco and particularly to enter contracts with leaf-buying entities. Tobacco does seem to generate income for farmers in Indonesia, but the analysis of those who switched from tobacco between waves 1 and 2 indicates that non-tobacco farmers tended to fare better both in terms of income per hectare and labor hours dedicated to growing. Targeted small loans for non-tobacco economic activities—which could even occur within the parameters of public-private partnerships to mitigate risk for both parties—could play a crucial role in opening opportunities for these households. Recent innovations to expand credit access for small-scale farmers include timing the loans and repayments to the agricultural cycle [19,20], using flexible collateral arrangements [21], and linking credit with weather insurance [22]. A cautionary tale comes from a study in Kenya where a small loan program successfully helped farmers to switch from subsistence farming to farming export cash crops, only for the exporters to refuse to buy the crops due to a lack of certification [23]. The farmers ended up losing money and returned to growing what they had grown before the program.

What will also be important to consider is the access to markets for agricultural commodities, and contractual relationships with companies for other crops. Generally, the situation of smallholder farming is difficult due to the starting point of minimal or no capital and the lack of power in relation to the companies that control the supply chain [5,6,24,25,26]. It will take broader attention to the system of agricultural production for opportunities to be generated and become attractive for tobacco farmers, so that they permanently shift to another commodity or economic activity.

The environmental harms and labor-intensive nature of tobacco growing alone serve as important reasons for governments to pursue alternatives [27]. However, the key to mitigating these challenges lies in generating viable economic activities for farmers to shift away from tobacco leaf cultivation. Although there are numerous intersecting factors that contribute to the decision to shift in and out of, or more permanently away from, tobacco, we have identified some key features of this decision context. Access to credit and a lack of experience with and knowledge of cultivating other crops are two key features that can serve as entry points for intervention if there is to be a genuine commitment to finding economically viable alternatives.

## Figures and Tables

**Table 1:** Final composition of the second wave survey sample, 2017.

District	Current Tobacco Farmer	Former Tobacco Farmer	Total
Magelang	80	10	90
Temanggung	78	12	90
Bojonegoro	133	47	180
Jember	138	42	180
Lumajang	83	37	129
Total	512	148	660

Source: Authors’ calculation of wave 1 and 2 tobacco farmers survey.

**Table 2:** Tobacco farming status in 2016 and 2017.

Status	n	Percent
*Tobacco farmers in wave 1, 2016*		
Stayed current tobacco farmer	437	66.21
Shifted out of tobacco farming	55	8.33
*Former tobacco farmers in wave 1, 2016*		
Stayed former tobacco farmer	93	14.09
Shifted into tobacco farming	75	11.36
Total	660	100

Source: Authors’ calculation of wave 1 and 2 tobacco farmers survey.

**Table 3:** Thought of switching and actual switching behavior.

Whether or not Current Tobacco Farmers Considered Switching in 2016	Stayed Current in 2017	Shifted out of Tobacco in 2017	Total
Considered switching	26	8	34
%	76.47	23.53	100
Did not consider switching	411	47	458
%	89.74	10.26	100
Pearson Chi-squared test	$\chi^{2}$ = 5.611	*p*-value = 0.018	

Source: authors’ calculation of wave 1 and 2 tobacco farmers survey.

**Table 4:** Reasons stated during wave 1 survey on why farmers opted out of tobacco farming.

Opting out Reasons Stated by Former Tobacco Farmers, 2016	Stayed Former in 2017, %	Shifted into Tobacco 2017, %	Pearson Chi-Squared, *p*-Value
Low price	35.48	20.00	0.027
Unfair grading	3.23	1.33	0.424
Inability to sell crop	13.98	8.00	0.224
More attractive alternative	7.53	10.67	0.478
Effect on land	9.68	8.00	0.705
Relationship with contracting co	2.15	0.00	0.201
Weather (rain)	20.43	42.67	0.002
Capital	5.38	5.33	0.990
Observations (*n*)	93	75	-

Source: authors’ calculation of wave 1 and 2 tobacco farmers survey.

**Table 5:** Household characteristics and farming outcomes in wave 1, 2016.

Variables	Former Tobacco Farmers, 2016			Tobacco Farmers, 2016		
	A: Stayed Former, 2017	B: Shifted into Tobacco, 2017	Wilcoxon Rank-Sum Test, *p*-Value	C: Stayed Current, 2017	D: Shifted out of Tobacco, 2017	Wilcoxon Rank-Sum Test, *p*-Value
*Households characteristics in wave 1*						
Head of household (HH) age in years	50.46	49.67	0.648	50.00	49.93	0.799
HH tobacco farming experience in years	-	-	-	20.07	14.09	0.001
HH years of schooling in years	5.796	6.227	0.366	5.314	5.40	0.629
Household size	3.645	3.947	0.215	3.977	3.86	0.358
1 if contract tobacco farmer	-	-	-	0.185	0.218	0.558
1 if Jember	0.226	0.200	0.686	0.281	0.38	0.124
1 if Temanggung	0.0968	0.120	0.630	0.158	0.054	0.041
1 if Magelang	0.0860	0.133	0.326	0.160	0.04	0.014
1 if Bojonegoro	0.269	0.147	0.056	0.279	0.40	0.064
1 if Lumajang	0.323	0.400	0.299	0.121	0.13	0.898
Asset, in log	14.88	15.15	0.494	15.33	14.23	0.090
*Farming outcomes in wave 1*						
Land cultivated, hectare	13.79	7.493	0.994	12.23	4.24	0.087
Share of land for tobacco	-	-	-	38.21	29.92	0.017
Share of land for non-tobacco	88.20	89.30	0.814	61.30	66.40	0.095
Yield in kg, tobacco	-	-	-	539.3	165.70	0.000
Tobacco sales, PPP	-	-	-	1276.3	391.40	0.000
Tobacco income, PPP	-	-	-	−224.1	20.53	0.6973
Crop sales dry season, PPP	2402.3	2518.5	0.776	308.6	280.60	0.666
Crop income dry season, PPP	1448.0	1361.6	0.703	174.8	148.40	0.669
Crop sales wet season, PPP	1569.9	2644.1	0.186	1614.7	586.90	0.005
Crop income wet season, PPP	914.0	1939.8	0.109	711.8	1.94	0.068
Agricultural wage income, PPP	375.3	533.4	0.259	354.6	534.80	0.233
Non-agricultural wage income, PPP	1800.0	1953.0	0.127	1421.2	1451.00	0.493
Household labor hours	366.9	393.0	0.831	817.6	714.70	0.066
Income per hectare, PPP	10,332.2	12,010.1	0.013	−1292.2	1703.50	0.904

Source: Authors’ calculation of wave 1 and wave 2 survey data. The term PPP refers to purchasing power parity.

**Table 6:** Determinants of shifting in-and-out of tobacco farming, average marginal effects.

Dependent Variable: Farming Outcomes	A: Stay Former	B: Into Tobacco	C: Stay Current	D: Out of Tobacco
Income per hectare (US$1000 PPP), wave 1	0.000357	0.00102 **	−0.00137	−0.00000722
	(0.000632)	(0.000516)	(0.000944)	(0.000640)
Rainfall deviation, wave 1	−0.115	0.0397	0.330 *	−0.254 **
	(0.159)	(0.157)	(0.189)	(0.124)
1 if have tobacco farming contract	−1.358 ***	−0.968 ***	2.029 ***	0.297 ***
	(0.138)	(0.118)	(0.133)	(0.0474)
log of agricultural wage income, wave 1	−0.000702	−0.0000320	0.000234	0.000500
	(0.00114)	(0.00100)	(0.00144)	(0.000987)
log of non-agricultural wage income, wave 1	−0.000396	0.000934	0.0000544	−0.000592
	(0.00113)	(0.00112)	(0.00157)	(0.00106)
log of asset, wave 1	−0.00259	−0.00186	0.00856	−0.00410 *
	(0.00397)	(0.00268)	(0.00535)	(0.00213)
log of cultivated area, wave 1	−0.0155 ***	−0.0112 ***	0.0311 ***	−0.00431
	(0.00408)	(0.00319)	(0.00542)	(0.00318)
HH age	0.000105	−0.0000349	0.000399	−0.000468
	(0.00146)	(0.00113)	(0.00172)	(0.00114)
HH schooling	0.00673	0.00950 **	−0.0158 ***	−0.000454
	(0.00493)	(0.00421)	(0.00594)	(0.00357)
HH size	−0.0154	0.000271	0.0155	−0.000348
	(0.00940)	(0.00884)	(0.0125)	(0.00888)
Observations	638	638	638	638
Controls	Yes	Yes	Yes	Yes
Robust Standard Error	Robust	Robust	Robust	Robust

Source: authors’ calculation of wave 1 and 2 survey data. Note: Numbers in parentheses are standard errors. The signs *, **, *** indicate statistical significance at 10, 5, and 1%, respectively. Estimated average marginal effects for the controls such as marital status, district dummies, and a dummy for missing profit per area are not shown for brevity. We keep households whose income per hectare is within the 1st and 99th percentile.

**Table 7:** Sample Quotes from Farmers from FGDs.

Theme	Sample Farmer Quote
Relationship with tobacco companies	*“The main problem is there is only one buyer here.”* (Lumajang) *“The middleman doesn’t pay right away… only when he is paid by the factory.”* (Jember)
Profitability	Profitable (Referring to the 2017 harvest): *“it was the best year. Some was 80 K. My harvest was also 80 K. it was the most expensive ever.”* (Lumajang) Not profitable (referring to the 2016 harvest): *“We just left it behind because we do not know who wants to buy it. No one wants to buy it. That’s the problem. We can plant tobacco. We have harvested it. But, there is nobody wants to buy it. So, it was left behind until it becomes damaged. On contrary, when we plant chili, though the price falls away, it is still in demand in the market.”* (non-contract farmer from Mediunan)
Credit/Capital	Lack of credit from other sources as a reason for taking on contracts with tobacco companies: *“…suppliers do not want to give loans because if the farmers suffer from fail, they indeed do not want it to happen or to buy the tobacco.”* (Sugigwaras) Lack of access to capital for non-tobacco economic activities: *“Of course I want to develop my shop, but I do not have enough capital.”* (Mediunan)
Weather	*“Tobacco is the only crop that thrives in the tobacco-growing (dry) season”* (Bojonegoro) (Responding to the top two reasons they grow tobacco): *“First of all, the weather. Second, it is already tradition in this Bades village”* (Lumajang)
Culture/Tradition	*“For me, I would take the risk. My land is located in lower ground so I do not worry about the plants to die. That’s why I keep planting tobacco. When it is time to plant tobacco, I plant tobacco. Come rain or shine. It is my tradition.”* (Bojonegoro)
Information	Lack of information: When asked where they get information on tobacco leaf prices: *“Just from the buyers”* (Magelang) When asked if they receive information about weather or climate from the government’s Meteorology, Climatology and Geophysical Agency (BMKG): *“No, never. Just from TV.”* (Lumajang) Successful information sharing: *“In the early planting season, I am always invited by plantation service [authors’ note: this service is governmental] to get information about the weather from BMKG. And then I share the information to the other farmers. In 2016, there has been a warning that the weather is wet so that I informed it to the farmers.”* (Temanggung)

## Appendix A. Guiding Questions and Concepts for Focus Group Discussions

### Appendix A.1. Current Farmers

- How much can you say do you know the tobacco industry (actors—buyers and sellers, where the tobacco leaves go and who use/consume them)?

If farmers sell to middleman, ask farmers whether they know whom the middleman sell the tobacco to.

If farmers sell directly to warehouse or company collectors ask which company.

How well do they know about the market of other crops?

- What is the general experience in tobacco farming? What are the most important issues for farmers?
  ○ Probe their views on pricing (how price is determined, who make the decision on the selling price of tobacco).

Price in the past few years. Are they planning their tobacco planting activities based on previous prices? How accurate are previous prices to help them plan their next planning season? How did they use past prices (e.g., how many years going back and expectation of future prices) to plan their planting season?

Who has more bargaining power on the price decision?

○ Probe their view on inputs (how do they obtain input, how do they pay-cash, loan, is input considered in determining price of tobacco).
○ Is there any difference between contract and non-contract farmer regarding pricing and inputs?

Describe the contract term and condition, obligation and penalties

○ Probe income fluctuation (what factors cause fluctuation).
○ Do they sometime keep the land fallow or engage in crop rotation to keep the soil productive in the long run?
○ Do you experience fluctuation in other crops also?
○ Probe loans/debts (these are for clarification and follow-up, if they do not come up in the discussion).

Probe rent vs. mortgage issue

- How profitable is tobacco farming?
  ○ How profitable tobacco farming compared to other crops?
  ○ Probe how do they compute net gain, and account for the labor involved in farming, including their own labor (these are for clarification and follow-up, if they do not come up in the discussion).
  ○ Negative profit: is it the way they calculate input cost or was last year especially bad year?
  ○ How was your profit last year compared to last year in tobacco farming? How about the profit from other crops?
- How important is tobacco farming for them, relative to other economic activities?
  ○ Probe the relative contribution of other activities to the household income?
  ○ If not profitable why they still farm tobacco.
  ○ What other crops are you growing aside from tobacco? Why?
  ○ What they think the impact of tax increase on their business?
- Have they considered shifting to other crops and leaving tobacco faming?
  ○ Probe why.
  ○ What other crops?
  ○ Do you think your children will continue working in tobacco planting activities in the future? Probe why
  ○ If not how do you prepare them?

Other topics:

- Labor exchange between farmers in the same village.
- Division of labor (participation in the tobacco farming) by age?
- Do you have free time to do more leisure or other activities?

Closing Question:

To close out our discussion, please think about your general experience in tobacco farming and tell us your expectation to the future of tobacco farming

### Appendix A.2. Former Tobacco Farmers

- Experience in tobacco farming?
  ○ Why you left tobacco farmin—what factors made you to decide leaving tobacco farming?
  ○ Have you completely switched or still on off farming tobacco; if completely switch why; if still off and on why?
  ○ Are you better off now compared to when still farming tobacco?
  ○ When you were still doing tobacco farming what was the contribution of tobacco income to your total income?
  ○ Do you take less loan now than before?
  ○ Do you feel more food secure now than before?
  ○ Did household food security factor at all into your decision to switch?
  ○ Do you have more time now to do economic, leisure or other activities?
  ○ What do you do with input items purchase for tobacco farming
  ○ What factors would make you switch back to tobacco farming
- Impact of increase in taxation
  ○ What do you think the impact of tax increase on tobacco farming?
  ○ If there were increase in the selling price of tobacco would you consider shifting back to farming tobacco?
  ○ What factors would make you switch?

Do you feel you now have more time compared to when you were farming tobacco? What do you use your extra time if you do?

Did labor division change? why?

Closing Question:

To close out our discussion, please think about your general experience in tobacco farming and tell us your expectation to the future of tobacco farming.
